# Do chimpanzees anticipate an object’s weight? A field experiment on the kinematics of hammer-lifting movements in the nut-cracking Taï chimpanzees

**DOI:** 10.1007/s10071-017-1144-0

**Published:** 2017-12-01

**Authors:** Giulia Sirianni, Roman M. Wittig, Paolo Gratton, Roger Mundry, Axel Schüler, Christophe Boesch

**Affiliations:** 1 Max Planck Institute for Evolutionary Anthropology, Deutscher Platz 6, 04103 Leipzig, Germany; 2 Taï Chimpanzee Project, CSRS, BP 1303, Abidjan, 01 Côte d’Ivoire; 3 Institut für Angewandte Trainingswissenschaft, Marschnerstraße 29, 04109 Leipzig, Germany

**Keywords:** Kinematics, Motor cognition, Tool use, Chimpanzees, Camera traps, Weight

## Abstract

When humans are about to manipulate an object, our brains use visual cues to recall an internal representation to predict its weight and scale the lifting force accordingly. Such a long-term force profile, formed through repeated experiences with similar objects, has been proposed to improve manipulative performance. Skillful object manipulation is crucial for many animals, particularly those that rely on tools for foraging. However, despite enduring interest in tool use in non-human animals, there has been very little investigation of their ability to form an expectation about an object’s weight. In this study, we tested whether wild chimpanzees use long-term force profiles to anticipate the weight of a nut-cracking hammer from its size. To this end, we conducted a field experiment presenting chimpanzees with natural wooden hammers and artificially hollowed, lighter hammers of the same size and external appearance. We used calibrated videos from camera traps to extract kinematic parameters of lifting movements. We found that, when lacking previous experience, chimpanzees lifted hollowed hammers with a higher acceleration than natural hammers (overshoot effect). After using a hammer to crack open one nut, chimpanzees tuned down the lifting acceleration for the hollowed hammers, but continued lifting natural hammers with the same acceleration. Our results show that chimpanzees anticipate the weight of an object using long-term force profiles and suggest that, similarly to humans, they use internal representations of weight to plan their lifting movements.

**Electronic supplementary material:**

The online version of this article (10.1007/s10071-017-1144-0) contains supplementary material, which is available to authorized users.

## Introduction

Many animal species rely on object manipulation to accomplish tasks crucial for their survival, particularly in the context of food retrieval and processing. Animals use different grasping organs (e.g., trunk, tongue, mouth, hands) to select, pick, transport and process food items, with a few species (especially among primates, but also elephants and birds) notable for their manipulative skills (e.g., Hayashi 2015; Martin and Niemitz 2003; Parker 1974; Rutz et al. 2016). The pattern and complexity of object manipulation is thought to reflect the level of cognitive development as well as that of manual control (Byrne 2001; Hayashi 2015).

Weight has paramount importance in determining how animals establish efficient interactions with physical objects, as it determines the grip and lifting force required (Johansson and Flanagan 2009). An object’s weight can be directly assessed by kinesthetic feedback, in which the muscular effort required to move and lift the object is processed by the nervous system (Robinson 1964). Kinesthetic perception of weight has been investigated in a handful of non-human animals (great apes: McCulloch 1941; Schrauf and Call 2011; Schrauf et al. 2012; capuchin monkeys: Schrauf et al. 2008; Visalberghi and Néel 2003; Visalberghi et al. 2009; certain seed-harvesting birds: Heinrich et al. 1997; Langen 1999). It has been shown that, at least in humans and chimpanzees, kinesthetic feedback perceived during movement is stored in short-term motor memory and can be recalled during repeated manipulations of the same object (Kent 2006; Povinelli 2012). In addition, humans are known to anticipate the weight of an object prior to any kinesthetic interaction with it: by using a cluster of visual stimuli (e.g., size, texture, color), our brains can recall from memory an anticipatory representation of the weight of an object we have never interacted with before (Buckingham et al. 2009; Gallivan et al. 2014; Gordon et al. 1993; van Polanen and Davare 2015). This representation (described as a ‘long-term force profile’ by Povinelli 2012 and as an ‘internal model’ by Krakauer and Shadmehr 2006) is formed through a generalization of repeated previous experiences with similar objects and is rapidly updated using kinesthetic information acquired during the movement itself if the predicted weight does not match the subsequent perception (Flanagan and Beltzner 2000; van Polanen and Davare 2015). Long-term force profiles have been proposed to be necessary for dexterous object manipulation by ensuring a stable grasp, determining speed and precision in execution and producing a smooth lift, as kinesthetic feedback mechanisms alone are generally too slow (Johansson and Flanagan 2009; Johansson and Westling 1984, 1988; van Polanen and Davare 2015).

The use of tools is regarded as one of the most sophisticated forms of object manipulation, as tool users must establish a dynamic spatial relation between at least two objects (Fragaszy et al. 2004; Hayashi 2015; Matsuzawa 1996). Tool use allows for a more efficient exploitation of available resources (Boesch and Boesch 1993; Möbius et al. 2008; Shumaker et al. 2011; Tebbich et al. 2002), with the selection of a suitable tool and its precise manipulation drastically influencing the overall outcome (Fragaszy et al. 2010; Luncz et al. in press). It is thus not surprising that the ability to form internal representations for object weight is well documented in humans, the species with the most outstanding technological achievements, and it seems likely that the same ability is found in animals that routinely engage in object selection and skillful object manipulation. Chimpanzees, one of our closest living relatives, are indicated by some authors to share with humans the cognitive machinery for dealing with their physical world (e.g., Boesch and Boesch 1990; Goodall 1970; Tomasello and Herrmann 2010). In the wild, all known chimpanzee populations have been observed to use and manufacture tools, showing a diverse and highly complex repertoire of tool use (McGrew 2010). Surprisingly, despite enduring interest in tool use in chimpanzees, as well as other non-human animals (Sanz et al. 2013), there has been very little investigation of their ability to form long-term force profiles to derive expectations about an object’s weight from visual cues.

Hanus and Call (2008) have shown that captive chimpanzees can infer the location of a food item based on the effect it exerts upon a balance and thus use a dynamic visual stimulus to identify the heavier of two objects. However, these authors did not directly investigate whether chimpanzees, like humans, form long-term force profiles and apply them when interacting with objects. Typically, studies on the anticipation of weight in humans address the appreciation of a relationship between size and weight by asking experimental subjects to lift objects of different sizes but the same weight, and measuring lifting (or grip) forces and/or lifting accelerations (e.g. Gordon et al. 1991; Flanagan and Beltzner 2000; Rabe et al. 2009). In this type of experiment, anticipation of weight based on size is revealed by an excess of force/acceleration in grasping/lifting the larger object compared to the smaller object (‘overshoot’), which tends to disappear after a few trials as the subjects become familiar with the actual weights. To our knowledge, the only study of this kind in non-human animals was conducted by Povinelli (2012) and did not provide conclusive results. In that study, laboratory chimpanzees were trained to displace two boxes of different sizes but the same weight and the maximum height of each displacement was measured. As predicted by the weight anticipation hypothesis, chimpanzees lifted the larger box higher than the smaller box. However, this difference persisted in subsequent trials, after chimpanzees had obtained kinesthetic information about the actual weights of the boxes. The latter result suggests that the difference in box size acted as a confounding variable in this experiment. While human subjects can be asked to lift objects according to the experimenter’s requirements, Povinelli’s (2012) chimpanzees were performing a non-goal-oriented task, and they might have been motivated to lift the larger box higher regardless of their anticipation of its weight. Even more importantly, the power of this study might have been limited by the choice of height (i.e., the final result of the movement) as the index of a subject’s expectations. In fact, in humans, expectations about object weight become visible in their motor output during the initial phase of lifting, in the form of early peaks in the lifting force (Flanagan and Beltzner 2000). Finally, Povinelli’s (2012) experiments involved captive chimpanzees which had to familiarize themselves with a limited set of unusual objects before using them in a non-goal-oriented task, but the ability to form ‘long-term force profiles’ via generalization of past experiences is probably better investigated by observing animals interacting with objects used in daily life that belong to a well-defined functional category, including thousands of individual objects that are, or could be, used in a goal-oriented and ecologically relevant routine activity.

In the present study, we proposed a new test of whether chimpanzees use internal representations (long-term force profiles) to anticipate the weight of novel objects of a known category based on their size. We tackled the main weaknesses of previous studies by performing a field experiment, involving wild chimpanzees dealing with objects of a highly familiar functional category that are lifted to fulfill a well-determined goal, and using camera-trap video recordings to measure lifting accelerations.

Chimpanzees (*Pan troglodytes verus*) of the Taï forest, Côte d’Ivoire, habitually crack open several species of hard-shelled nuts by placing them on anvils (hard roots, stones or branches within a tree) and pounding them with wooden or stone hammers (Boesch and Boesch 1982; Visalberghi et al. 2015; weight range 0.2–15 kg). Nut-cracking has been described as one of the most complex forms of tool use in non-human animals, as it implies the establishment of two spatial relationships among three objects (hammer, nut and anvil), requires bimanual (and sometimes foot) coordination and a high level of motor control (Boesch and Boesch 1993; Bril et al. 2009; Matsuzawa 1996). The nut-intake rate is influenced both by the number of strikes needed to open a nut and by the precision of hammering (Luncz et al. in press; Sirianni et al. 2015). Hammer weight clearly influences the efficiency of a nut-cracking session (Boesch et al. 2017; Fragaszy et al. 2010; Luncz et al. in press), and chimpanzees have been shown to be sensitive to the functionality of this physical property by selecting for hammer weight (laboratory experiments: Schrauf et al. 2012; field observations: Boesch and Boesch 1982, 1984; Luncz et al. 2012), and by doing so in a sophisticated, conditional way (assessed in wild chimpanzees by Sirianni et al. 2015). Nut-cracking movements by wild chimpanzees thus appear to be an ideal model for investigating the anticipation of weight in non-human animals.

We performed our field experiment in the Taï forest, providing wild chimpanzees with nut-cracking hammers of a familiar material commonly available in the forest litter (*Coula* wood). All hammers were of the same size. Some hammers, however, were artificially hollowed so as to be lighter than natural, solid hammers.

We compared lifting accelerations for the modified (hollowed) and natural (solid) hammers, predicting that, if chimpanzees anticipate the weight of hammers based on their size, they should initially (when they lift each hammer for the first time) apply a similar force to the solid and the hollowed hammers, which would result in a higher acceleration for the latter type (‘acceleration overshoot’). Moreover, we predicted that, as chimpanzees learn about the actual weights of the two hammer types (e.g. after cracking one nut open), they would continue to apply the same force (and hence obtain the same acceleration) to natural hammers, but would instead ‘tune down’ the force used for hollowed hammers (lighter than expected), so that the acceleration overshoot would tend to fade away. Indeed, observing such a ‘tuning down’ for hollowed hammers, but not for natural hammers, would rule out the only possible alternative explanation for the initial overshoot (i.e., that chimpanzees apply a fixed ‘standard’ force when they first lift a hammer, regardless of its appearance and size), thus conclusively demonstrating anticipation of weight and long-term force profiles in chimpanzees.

## Methods

### Study site and period of data collection

We collected behavioral data during field experiments on habituated chimpanzees from the North Community of the Taï National Park, Côte d’Ivoire. Data were collected by GS from late November 2012 to February 2013 on a total of 16 individuals of all age classes (Table 1).

**Table 1:** Number of observations per individual, hammer type (*H* hollowed, *N* natural) and nut order

Name	Sex	Age	First nut		Second nut	
			H	N	H	N
Bartok	m	8	2	3	2	1
Belle	f	37	0	2	2	5
Faust	m	14	2	4	7	9
Mandela	m	12	2	1	5	13
Massa	m	7	0	4	1	4
Mystere	f	38	0	1	2	7
Naomi	f	9	3	2	2	3
Narcisse	f	29	0	0	1	1
Nimba	m	6	0	2	0	1
Noureyev	m	16	0	0	4	5
Pandora	f	17	2	2	3	4
Pastis	m	7	2	2	3	2
Perla	f	37	1	1	3	3
Porthos	m	12	6	5	12	11
Surprise	f	15	0	0	1	2
Volta	f	17	0	0	5	4
Total			20	29	53	75

The number of observations retained for analyses is reported. For each individual, the age in years as of Nov 2012 is given

### Lab-sites

**Fig. 1:**
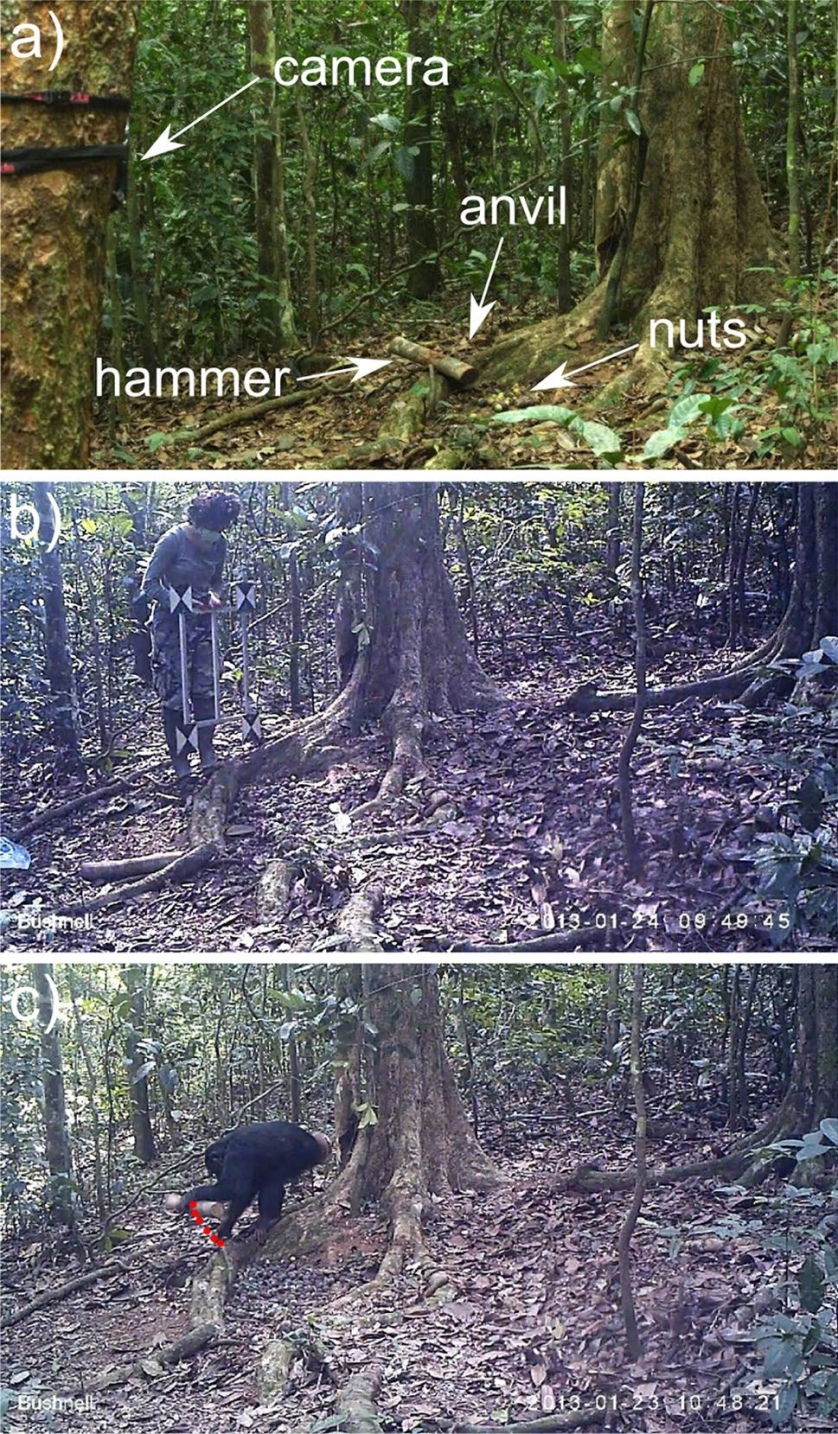
**a** A typical lab-site: a camera trap attached to a tree trunk, with an open view of a root anvil with an experimental hammer and nuts. **b** GS is videotaping the calibration device, orienting it in different positions; this picture shows the orientation selected as the best approximation of the plane along which the hammer-lifting movement occurred in (**c**). **c** The red dots show the position of the hammer at each frame, during the lifting movement. The composite figure was created in Inkscape 0.91

Eight lab-sites (Fig. 1a) were set up at *Coula edulis* trees in the territory of the Taï north group community. Each lab-site contained a root anvil (on which chimpanzees were previously observed to spontaneously crack nuts), a camera trap (previously calibrated, see ‘Kinematic measures’ section), *Coula edulis* nuts, and one experimental hammer (see ‘Hammers’ section). Experiments were set up in the absence of chimpanzees. For additional information on lab-site maintenance, nut provisioning and hygienic rules undertaken in order to prevent pathogen transmission to chimpanzees, see Online Resource 1.

Each lab-site was monitored by a Bushnell Trophy Cam HD Max 119476 camera trap, which provided automatic video recordings of experimental sessions at 1280×720p resolution and with frame rate optimized according to the available light (15–20 frames per second). Each camera was secured to a tree trunk 5–10 m from the anvil.

### Hammers

**Fig. 2:**
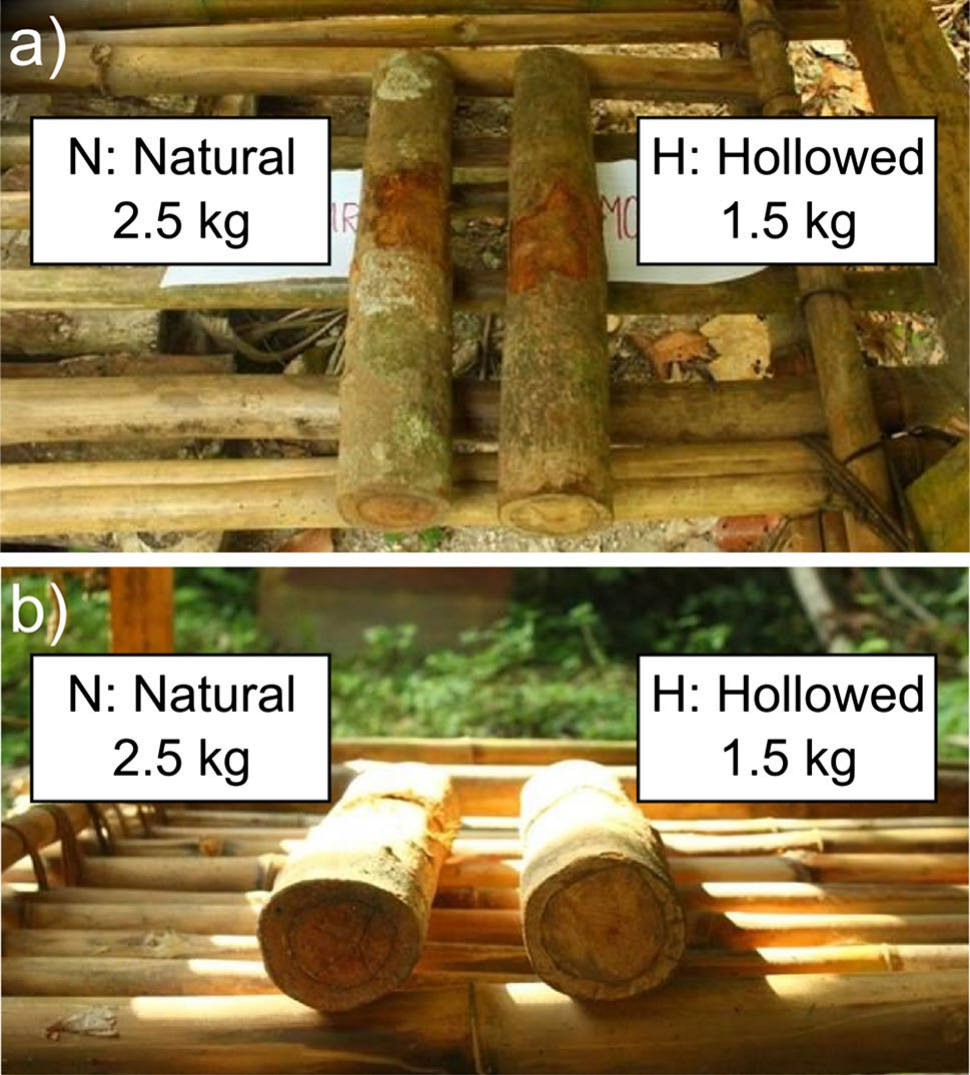
Hollowed (H) and natural (N) hammers. The hollowed modified and solid natural hammers were of the same size and external appearance (the slight variation in color in the two photographed hammers reflects the natural bark-color variation of fresh *Coula edulis* wooden clubs), but different weight (H was lighter than N). Both hammers in these photographs showed usage wear (**a**). The circular marks at the extremity of each hammer (**b**) are the real and fake plugs of the H and N hammer, respectively (see ‘Methods’ section ‘Hammers’). The composite figure was created in Inkscape 0.91

During the course of the field experiment, we employed a total of 22 hammers, belonging to two types: natural (N) and hollowed (H). All hammers consisted of approximately cylindrical sections of *Coula edulis* branches of ca. 7 cm diameter and ca. 50 cm length (Fig. 2a). The eleven natural hammers (N) were solid, weighing ca. 2.5 kg each. Eleven hollowed hammers (H) were created by a professional carpenter in Abidjan (Côte d’Ivoire), by removing the core material from natural hammers, thus reducing their weight to ca. 1.5 kg. Plugs obtained from the same piece of wood were glued to close the holes produced at the two ends of hollow hammers. Fake plugs were added to natural hammers too, to minimize the chance that chimpanzees could identify the hollowed hammers via visual or olfactory cues (Fig. 2b). The use of a relatively large number of hammers (N = 11 of each type) allowed us to run multiple lab-sites simultaneously as well as avoid pseudo-replication.

One hammer at a time was presented at each lab-site (Fig. 1a). All hammers were individually marked with a number carved at their cut end, such that they could be identified even when chimpanzees moved them across lab-sites. After each chimpanzee visit, we removed the hammer (if still present) from the lab-site and replaced it with another random hammer, so that both individual hammers and hammer types were frequently swapped across lab-sites.

### Kinematic measures

Before and after the chimpanzees used a lab-site, we videotaped a calibration device. The device consisted of a 50 × 55 cm rectangular metal frame, whose corners were marked with contrasting colors so as to be easily identifiable in video recordings (Fig. 1b). Since we could not anticipate the exact position that chimpanzees would take up when using the hammers and anvils, we videotaped the calibration device in different orientations, in order to be able to later select the orientation closest to the plane in which the movement of the hammer had occurred (Fig. 1b, c).

Mess2D software, developed by the Institut für Angewandte Trainingswissenschaft (IAT, Leipzig), was used to calibrate video recordings by translating the position of each pixel into two-dimensional spatial coordinates describing the selected plane (*x* and *y* for the horizontal and vertical axes, respectively). A point-and-click user interface (also in the Mess2D software) was used to obtain a two-dimensional description of the lifting movement by recording the position of the hammer (more precisely, the contact point of the hammer with the skin between the index finger and the thumb of the chimpanzee’s hand) in each video frame (Fig. 1c). Recording of hammer position was started approximately 3–4 frames before the apparent onset of the lifting movement. Kinematic parameters were extracted using custom *R* scripts. Lifting movement was formally identified as beginning with the first of at least three consecutive frames with positive vertical displacement (i.e., for which *y*_i+1_ − *y*_i_ > 0) and ending with the frame corresponding to the next height maximum (i.e., for which *y*_i_ − *y*_i−1_ > 0 and *y*_i_ − *y*_i+1_ > 0). Maximum lifting acceleration (*a*_max_) was defined as the maximum difference between the vertical velocities calculated for two consecutive frame pairs during a lifting movement [*a*_max_ = max((*y*_i+1_ − *y*_i_) − (*y*_i+2_ − *y*_i+1_))].

### Data selection

We predicted that chimpanzees would show an ‘acceleration overshoot’ for the hollowed hammers (H) when they had no previous experience (see Introduction). Secondarily, we were interested in the possible reduction of this overshoot after chimpanzees had gained experience with a specific hammer. Therefore, we selected two sets of data, representing ‘naïve’ (before any direct kinesthetic interaction with a specific hammer) versus ‘experienced’ (after interaction with the hammer) conditions. For the naïve condition, we analyzed the first lifting movement that resulted in a direct strike at the first nut in a nut-cracking episode (we defined an episode as a sequence of nuts cracked by an individual chimpanzee at a given lab-site on a given day with a given hammer). All first lifting movements that were preceded by physical interactions from which chimpanzees could have gathered information about the actual weight of a hammer before using it (e.g., displacement or dragging of the hammer) were excluded from the naïve condition. For the experienced condition, we selected all first lifting movements that resulted in a directed strike at the second nut in a nut-cracking episode. We only considered the first lifting movement for each nut because previous studies of chimpanzees nut-cracking showed that subsequent hammering movements (including lifts and downward hitting) in a sequence directed to the same nut are kinematically different from the first, with their features depending on the outcome of the previous hammering (Bril et al. 2009). Therefore, for the interests of our study, only the firsts lift of each sequence were directly comparable to each other. All lifting movements for which it was not possible to compute reliable kinematic parameters due to low visibility (e.g., poor light or obstructed view) were excluded from both conditions. For examples of video recordings that were discarded or retained, see Online Resources 2, 3 and 4.

### Statistical analyses

We tested the prediction that chimpanzees lifted the hollowed hammers (H) with a higher acceleration than when lifting natural hammers (N) (‘acceleration overshoot’) using a linear mixed model (LMM, Baayen 2008), with maximum vertical acceleration (*a*_max_) as the response and including ‘hammer type’ (H vs. N) as a predictor. Within the same LMM, we also tested for a reduction in the acceleration overshoot from the ‘naive’ to the ‘experienced’ condition by including the interaction between the predictors ‘hammer type’ and ‘nut order’ (first vs. second nut, coded as zero and one). We also controlled for the effect of chimpanzee age (in years) and for the random effect of individual subject and nut-cracking episode. To keep type I error rates at the nominal level of 5%, we included random slopes for hammer type (manually dummy coded and then centered), nut order and their interaction as well as for age within subject, but did not include the correlation parameters among random intercepts and random slopes terms (Barr et al. 2013; Schielzeth and Forstmeier 2009). Prior to the analysis, we square-root-transformed maximum acceleration to achieve an approximately symmetrical distribution and avoid potentially influential cases. We checked for the assumptions of normally distributed and homogeneous residuals by visually inspecting a qq-plot and the residuals plotted against fitted values (Quinn and Keough 2002), neither of which indicated any obvious deviations from these assumptions. The sample size for this model was 177 accelerations by a total of 16 individuals during 135 episodes (see also Table 1).

To rule out that chimpanzees could identify the artificially hollowed hammers (H) using visual and/or olfactory cues, we compared the frequency with which one exploratory behavior, sniffing (for an example, see video in Online Resource 5), that we could consider as intentional, was directed at each type of hammer before lifting a hammer for the first time in each episode (see above for the definition of an episode). We predicted that if chimpanzees could identify the hollowed hammers (H), they would be more suspicious toward these and exhibit a higher frequency of exploration than toward the natural hammers (N). To test for a difference between the two hammer types, we fitted a generalized linear mixed model (GLMM; Baayen 2008) with binomial error structure and logit link function, with presence of sniffing as the response and hammer type (H vs. N) as the test predictor. We controlled for the fixed effect of subject age and for the random effect of subject. In this model, we could not include random slopes for hammer type and age within subject, because the model failed to converge otherwise. The sample size was N = 115 approaches of a chimpanzee to a hammer, for a total of 16 individuals, with sniffing observed in 7 occasions. We found that chimpanzees did not obviously explore (i.e., sniff) the hollowed (H) hammers more frequently than the natural (N) hammers of same size and similar external visual appearance (estimate = 0.78; χ^2^ = 0.93, *df* = 1; *P* = 0.34).

Models were fitted in R (version 3.1.0, R Core Team 2014) using the functions lmer or glmer (package ‘lme4’ version 1.1-12, Bates et al. 2014). P values for individual effects were based on likelihood ratio tests comparing full models with reduced models (Barr et al. 2013; R function drop 1). We obtained confidence intervals for model estimates by bootstrapping (1000 replicates), using the function bootMer of the package lme4. Variance Inflation Factors (VIF, Field 2005) from standard linear models excluding the random effects showed no sign of collinearity among predictors (all VIF < 1.02; R function vif, package ‘car’, Fox and Weisberg 2011). We checked for model stability by excluding individuals one at a time from the data and fitting the same models to these subsets, which showed no indication of the presence of influential individuals.

The dataset produced and analyzed during the current study is available from the corresponding author on request.

## Results

We recorded a total of 266 clearly visible lifting movements that resulted in a direct strike to the first (N = 159) or the second (N = 128) nut in a nut-cracking episode. We discarded 89 first nut lifts that were preceded by a physical interaction between the subject and the hammer (see ‘Methods’ section), leaving us with 177 usable data points (Table 1).

The mean duration of lifting movements was 0.36 s (median = 0.35 s), corresponding to 6.22 (median 6) video frames. Maximum acceleration was observed on average 0.14 s (median = 0.12 s) after the start of the lift, corresponding to 2.36 frames (median = 2).

**Fig. 3:**
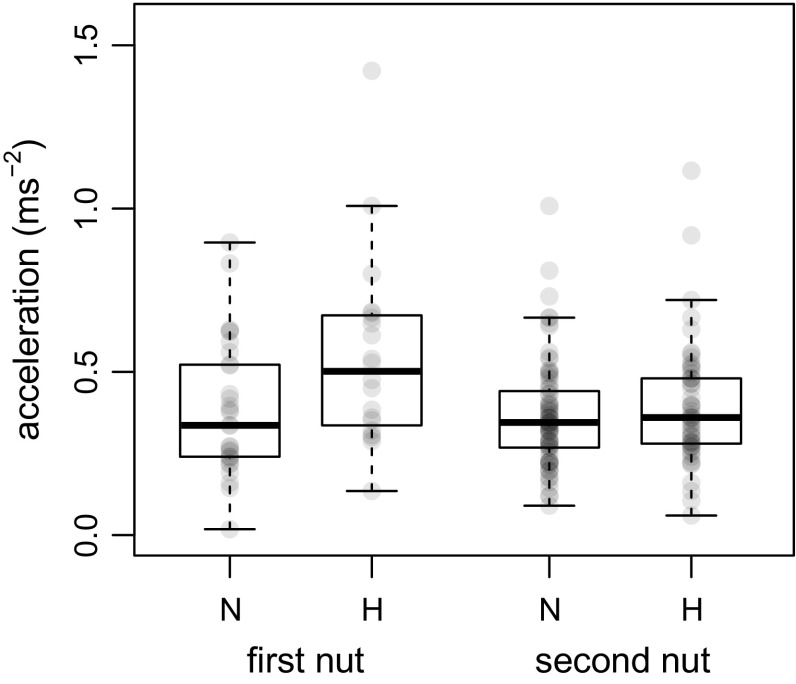
Maximum lifting accelerations for natural (N) and hollowed (H) hammers in the ‘naïve’ (first nut) and ‘experienced’ (second nut) conditions. Boxplots are superimposed upon raw data (semi-transparent filled circles). Plot was created in R

In the ‘naïve’ condition (first nut in a nut-cracking episode), the maximum acceleration of the first lift was higher for the hollowed hammers (H) than for the natural hammers (N), and the difference in acceleration between the two hammer types decreased markedly from the ‘naïve’ to the ‘experienced’ condition (second nut in a nut-cracking episode) (interaction between hammer type and nut order: χ^2^ = 4.72, *df* = 1, *P* = 0.03; Table 2; Fig. 3). The acceleration imposed upon the natural hammers was similar for first and second nuts (confidence intervals for ‘nut order’ encompassed zero; Table 2; see also Fig. 3), while the acceleration of hollowed hammers was significantly lower for second nuts than for first nuts (Table 2; Fig. 3).

**Table 2:** Summary of model results

Term	Estimate	SE	CI_lower_	CI_upper_	χ^2^	*df*	*P* ^a^
Intercept	0.613	0.039	0.541	0.690	^b^	^b^	^b^
Hammer type (H)	0.121	0.041	0.040	0.205	^b^	^b^	^b^
Nut order	0.014	0.031	− 0.044	0.079	^b^	^b^	^b^
Age	− 0.001	0.002	− 0.005	0.002	0.783	1	0.376
Hammer type: nut order	− 0.103	0.047	− 0.193	− 0.012	4.717	1	0.030

The table reports the estimated coefficient (Estimate) for each model term, with associated standard error (SE), lower and upper limits of the 95% confidence interval (CI_lower_, CI_upper_) and the likelihood ratio (χ^2^), degrees of freedom (*df*) and *P* value (*P*)

^a^ *P* vales and test results for individual predictors are derived from the R function drop 1

^b^ *P* values do not have a meaningful interpretation and are therefore not shows

## Discussion

In this study, we investigated chimpanzees’ anticipation of the weight of a nut-cracking tool from its size. Our results are consistent with the ‘acceleration overshoot’ predicted by the weight anticipation hypothesis, in that chimpanzees lifted the hollowed hammers with a greater acceleration than the natural hammers, in the ‘naïve’ condition (first nut in a nut-cracking episode) (Fig. 3). An acceleration overshoot for the lighter (hollowed) hammers in the ‘naïve’ condition would also be observed if chimpanzees, without any anticipation of hammer weight, initially lift any hammer with a similar force, regardless of its size. However, we also found that the acceleration of natural hammers did not differ between the ‘naïve’ and ‘experienced’ conditions (first vs. second nut in a nut-cracking episode), while the acceleration of hollowed hammers was lower in the ‘experienced’ than in the ‘naïve’ condition. The latter result can only be explained if chimpanzees accurately anticipated the weight of nut-cracking hammers based on their size. Therefore, our experiment conclusively showed anticipation of weight in chimpanzees and suggests that, similarly to humans, they use internal representations of weight (based on a generalization of previous experience) to plan their lifting movements. We can be confident that the chimpanzees could not visually distinguish hollowed hammers from natural hammers, as they showed no sign of a particular suspicion toward hollowed hammers (in that they did not sniff the hollowed hammers more often than the natural ones). This was consistent with the fact that GS, who manipulated the hammers daily during data collection, was never able to tell the two hammer types apart at a glance.

It has been suggested that the ability to anticipate the weight of an object based on long-term force profiles benefits humans by allowing quick and precise execution of movements (van Polanen and Davare 2015; Johansson and Flanagan 2009). However, in the context of chimpanzees’ nut-cracking behavior, the benefit of any long-term motor memory may be limited to the first few strikes with a given tool, as short-term motor memory (which chimpanzees have been shown to possess: Povinelli 2012) will quickly prevail during repeated use of the same tool. Indeed, the ability to generalize previous experiences to predict the weight of novel objects (long-term force profiles) can be of even larger advantage when the affordances of several objects must be quickly evaluated for prompt selection among many potential tools. In this respect, our results are consistent with our previous observation that Taï chimpanzees select in a sophisticated way for the weight of nut-cracking tools, very rarely engaging in prior explorative behaviors (Sirianni et al. 2015).

The physical basis of perceived heaviness has been extensively investigated in humans (e.g. Turvey et al. 1999), in which two main systems for processing and storing information regarding the force required to lift an object (weight) have been identified: a sensorimotor (muscular) and a purely cognitive (mentalistic) system (Flanagan and Beltzner 2000). The idea of a double encoding derives support from the neurobiology literature emphasizing that visual information is processed using distinct neural pathways depending on whether the information is used to control actions (sensorimotor) or make perceptual (cognitive) judgments (e.g. Goodale et al. 1991, 1994). Although the two weight-encoding systems generally work in concert, using the size-weight illusion paradigm, it was possible to show that they can also act independently (Flanagan and Beltzner 2000). In humans, this has been revealed by coupling behavioral measures of the sensorimotor path (i.e. the force of lifting movements) with direct interviews asking subjects about the expected weight of a particular object (Flanagan and Beltzner 2000).

However, chimpanzees cannot be asked about their weight expectations, and a behavioral approach is the only way to infer their mental processes. Therefore, our data do not allow us to distinguish between the cognitive or sensorimotor nature of the anticipation of object weight, a task well beyond the scope of our study. However, since the cognitive representation of weight by humans has been related to neural pathways which are also present in macaques (Flanagan and Beltzner 2000; Goodale et al. 1994), evolutionary parsimony suggests that cognitive representations of weight may exist in chimpanzees too.

Humans use more than apparent size to program lifting forces (e.g., object color or texture as clues to material and thus density; see Buckingham et al. 2009). While our experiments focused on hammer size (i.e., length, see Methods) as the visible feature used to recall internal representations of weight, it is likely that chimpanzees also employ a larger cluster of static visual stimuli to anticipate the weight of objects. In fact, wooden clubs used as hammers by Taï chimpanzees belong to several tree species and vary widely in terms of density, color and texture. Povinelli (2012) tested for modulation of lifting force based on the texture of artificial objects in captive chimpanzees, obtaining a negative result. However, the authors themselves acknowledged that the absence of a significant effect might have been due to the artificial nature of the objects used. Therefore, whether chimpanzees are able to anticipate the weight of an object by using color/texture as predictive visual cues remains an open question. Future experiments might investigate the ability of chimpanzees to use botanical skills to predict the weight of wooden clubs obtained from tree species possessing different average wood densities.

Apart from species-specific features, the actual density of any piece of wood in rainforest litter depends largely on its state of decay (water content, disintegration of fibers by fungi or termites, etc.). Although decay condition can be indicated by externally visible cues, any prediction of the density of wood comes with a degree of uncertainty, such that a significant mismatch between expected and perceived weight will often occur even when dealing with natural hammers. In such a context, prompt integration of kinesthetic feedback would allow for more precise control of the whole lifting movement. The weight anticipation hypothesis predicts that in the ‘naïve’ condition chimpanzees would, at least initially, apply exactly the same force to both natural and hollowed hammers in our experiment, as they were externally identical. However, humans are known to rapidly update their motor plan in cases where a mismatch occurs between the expected and the perceived weight, so that the lifting force applied to objects that are lighter than expected is tuned down within a few tenths of a second (e.g. Flanagan and Beltzner 2000). Likewise, while we found that maximum accelerations (*a*_max_) were greater for the lighter hollowed hammers than for the heavier natural hammers in the ‘naïve’ condition (Fig. 3), our measures do not seem to correspond to an identical lifting force being applied to the two hammer types (we obtained approximate measures of lifting force by adding the gravitational acceleration, *g* = 9.81 ms^−2^, to *a*_max_ and multiplying by the weight of the hammer, with or without an estimate of the contribution of the chimpanzee’s arms; see Online Resource 1 for details). In particular, the estimated maximum lifting force was still higher for the heavier natural hammers than for the lighter hollowed hammers (Fig. S2 in Online Resource 1). Our experiments relied on kinematic measures of a relatively low temporal resolution (limited by the 15–20 frames per second capture rate of our camera traps), and we recorded maximum vertical accelerations (*a*_max_) at, on average, 0.14 s after the onset of the lift. This implies that our measures likely reflect a mixture of anticipated motor program and adjustment based on perceived weight. Our results therefore suggest that, similarly to humans, chimpanzees quickly react to a mismatch between expected and perceived weight by integrating kinesthetic information acquired during the initial phase of lifting into an updated motor plan. The reaction time needed to adjust the lifting force when faced with a mismatch between expected and actual weight also seems to be similar in chimpanzees and humans. We observed that maximum lifting accelerations occurred on average within 0.14 s of the onset of the lifting movement, indicating that chimpanzees could have reacted to a perceived mismatch within such a short time. Similarly, experiments employing high-speed (400 Hz) force sensors (Flanagan and Beltzner 2000) have demonstrated that human subjects react to a weight mismatch within ca. 0.1 s and completely scale down to a ‘target’ force within ca. 0.2 s.

The use of camera traps to systematically study detailed aspects of behavior in wild animals remains restricted to only a few studies (De Moraes et al. 2014; Estienne et al. 2017; Filipczyková et al. 2017; Kühl et al. 2016; Musgrave et al. 2016; Otani 2001), with even fewer studies combining camera traps and field experiments (e.g., Gruber et al. 2009). To our knowledge, our study represents the first attempt to measure kinematic parameters of animal behavior from camera-trap recordings. Using camera traps allowed recording of multiple individuals simultaneously at several nut-cracking lab-sites, dramatically enlarging our sample size compared with that possible from a single human-operated camera. Indeed, nuts, hammers and anvils are abundant and dispersed throughout the Taï Forest, so that conditioning chimpanzees to spend a long time cracking nuts in a single spot would not be feasible (in contrast with Boa Vista capuchin monkeys, see Mangalam and Fragaszy 2015; Liu et al. 2016). Importantly, although we worked with habituated chimpanzees, the combination of field experiments and kinematic quantification from remote camera-trapping can, in principle, be extended to unhabituated populations and even elusive species. In conclusion, our study shows how camera-trap approaches can be used to detail subtle aspects of the motor planning process involved in nut-cracking behavior and demonstrated chimpanzees’ ability to accurately predict the weight of a tool in a natural context.

## Electronic supplementary material

Below is the link to the electronic supplementary material.

Online Resource 1. Supplementary Methods and Results (DOCX 244 kb)

Online Resource 2. Video material: a typical hammer lifting selected for the “naïve” dataset (i.e., a first lift directed to the first nut in a nut-cracking session not preceded by hammer manipulation) (AVI 41863 kb)

Online Resource 3. Video material: example of a hammer lift discarded from the “naïve” dataset due to heavy manipulation of the hammer (hammer throwing and transport) (AVI 116570 kb)

Online Resource 4. Video material: example of a hammer lift discarded from the “naïve” dataset due to a less intense manipulation of the hammer (hammer accidentally dropped from the anvil before using it) (AVI 78696 kb)

Online Resource 5. Video material: example of hammer exploration (sniffing) (AVI 377229 kb)
