# Supplementary material for: Do chimpanzees anticipate an object’s weight? A field experiment on the kinematics of hammer-lifting movements in the nut-cracking Taï chimpanzees
Source: Anim Cogn. 2017 Dec 1;21(1):109–18. doi: 10.1007/s10071-017-1144-0 (PMC5756265; doi:10.1007/s10071-017-1144-0)
Supplement: Supplementary file 1 — Online Resource 1. Supplementary Methods and Results (DOCX 244 kb) [file 10071_2017_1144_MOESM1_ESM.docx]

**ONLINE RESOURCE 1**

**Article Title:** Do chimpanzees anticipate object’s weight? A field experiment on the kinematics of hammer-lifting movements in the nut-cracking Taï chimpanzees

**Journal Name**: Animal Cognition

**Authors:** Sirianni Giulia^1^, Wittig Roman M^1,2^, Gratton Paolo^1^, Mundry Roger^1^, Schüler Axel^3^, Boesch Christophe^1^

^1^ Max Planck Institute for Evolutionary Anthropology, Deutscher Platz 6, 04103 Leipzig, Germany; ^2^ Taï Chimpanzee Project, CSRS, BP 1303, Abidjan 01, Côte d’Ivoire; ^3^ Institut für Angewandte Trainingswissenschaft, Marschnerstraße 29, 04109 Leipzig, Germany

**Corresponding author:** Giulia Sirianni [giulia_sirianni@eva.mpg.de](mailto:giulia_sirianni@eva.mpg.de)

**Supplementary Methods**

Maintenance of lab-sites:

Every second/third day or soon after a known chimpanzees visit to a lab area, we took care of the lab-site by providing fresh nuts, changing batteries of cameras and, after a chimpanzees visit had occurred, by replacing the SD card (videos were later downloaded in camp), making a new calibration (see section ‘calibration’, main text) and rotating hammers across lab-sites.

Nuts

Nuts were regularly collected in the forest and stored in camp on a dryer before being transferred to each lab-site. Each lab-site was provided with nuts after every second or third day or after any known chimpanzees visit, so that the anvil was left with at least 30 nuts. Nuts were placed on the side of the anvil and, during the 2012/2013 season when H and N hammers were tested, one nut was also positioned right on the anvil pit (*i.e.,* a shallow depression due to wear, by repeatedly cracking nuts) to reduce the probability of chimpanzees manipulating the hammer (e.g., displacement) before the first striking movement actually occurred (see Fig. S1) as much as possible.


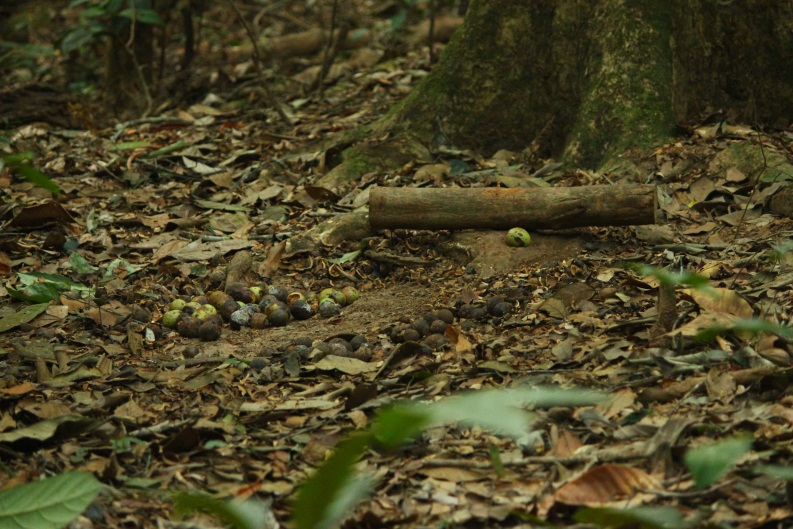


**Fig. S1** The experimental hammer was located on the anvil with a nut placed already in the anvil pit

Manipulation-storage-hygienic rules

To prevent pathogen transmission from humans to chimpanzees, together with the standard hygienic measures undertaken in the Taï Chimapanzees project (Grützmacher et al. 2017), we established a set of additional hygienic measures: 1) hands were disinfected prior any manipulation of hammers and nuts; 2) while handling nuts and hammers surgical masks and plastic gloves were used; 3) transported nuts (from collection sites to camp and from camp to lab-sites) were placed in disposable bean plastic bags; 4) before being transferred to the lab-sites collected nuts were stored for a few days on a dryer in direct sunlight at the periphery of the camp in order to use UV light as an additional disinfection step; 5) after the H and N hammers have been modified in Abidjan and transferred into the forest, they had been disinfected and put in quarantine for a week before being presented to the chimpanzees.

Comparing force applied to natural and hollowed hammers in the naïve condition

The weight anticipation hypothesis predicts that, in the 'naïve' condition, chimpanzees would, at least initially, apply exactly the same force to the natural and hollowed hammers in our experiment, as they were externally identical. To test whether differences between the recorded maximum accelerations for the natural (N) and hollowed (H) hammers corresponded to the application of identical forces, we 1) estimated the force applied in each hammer lifting and 2) tested for a difference between the two hammer types (N and H) in the naïve condition (first nut cracked within each nut-cracking episode) using Linear Mixed Models.

An accurate estimation of the effective contribution of the weight of chimpanzees' arm (or arms, whenever the hammer was lifted using both hands) to the lifting force would require a precise 3-dimensional reconstruction of the movement, which was beyond the technical limits of our procedure. Therefore, we calculated maximum lifting force for two extreme scenarios (such that the real values fell somewhere in between). The first estimate ignored the weight of the chimpanzee arm(s), i.e*.,* *F* = (*a*_max_ + *g*) * *hammer weight* (where *g* is the standard gravitational acceleration of 9.81 m/s^2^); the second estimate assumed that the whole arm behaved as a rigid body rotating about the shoulder (as in Günther and Boesch 1993), *i.e.,* *F* = (*a*_max_ + *g*) * (*hammer weight* + *w*_U_ * *L*_U_/*L*_TOT_ + *w*_F_ * *L*_F_/*L*_TOT_ + *w*_H_), where *w*_U_ is the weight of the upper arm, *w*_F_ is the weight of the forearm, *w*_H_ is the weight of the hand, *L*_U_ is the distance from the shoulder to the middle of the upper arm, *L*_F_ the distance from the shoulder to the middle of the forearm and *L*_TOT_ is the distance from the elbow to the middle of the hand. The approximate weight and length of each individual chimpanzee's arm segments (upper arm, forearm and hand) was calculated using the empirical equations proposed by Schoonaert et al. (2007). When the hammer was lifted using both hands (101 lifts, 57%), the estimated weight of the arm was doubled.

To test for differences between the two hammer types, we fitted two LMMs with Gaussian error structure, differing in the way the lifting force was estimated (either by ignoring the weight of the arm or by considering the arm as a rigid body rotating about the shoulder). In both models, maximum lifting force was the response variable, and hammer type (H or N) was the test predictor with fixed effect. We controlled for the effect of the age of the subject (in years) and for subject by including the corresponding fixed and random effect, respectively. To keep type I error rates at the nominal level of 5%, we included random slopes of hammer type (manually dummy coded and then centered) and age within subject, but not the correlation parameters among random intercepts and random slopes terms. Measures of force were square root transformed prior to running the model to achieve normally distributed and homogeneous residuals, which were visually checked by qq-plots and residuals vs. fitted values scatter plots, respectively. These analyses were limited to the naïve data set (N = 49 for a total of 12 individuals, see Table 1 in the main text).

**Supplementary results**

Under both approximations about the contribution of the weight of the arm to the lifting force, chimpanzees lifted the hollowed hammer (H) with a clearly lower force than the natural hammer (N) (Table S1, Fig. S2).

| **Table S1a.** Force estimated by ignoring the weight of the arm | | | | | |
| --- | --- | --- | --- | --- | --- |
| **Term** | **Estimate** | **SE** | **χ^2^** | ***df*** | ***P*** |
| Intercept | 5.055 | 0.011 | ^(1)^ | ^(1)^ | ^(1)^ |
| Hammer type (H) | -1.125 | 0.006 | 125.5 | 1 | < 0.001 |
| Age | -0.001 | 0.001 | 1.067 | 1 | 0.302 |

| **Table S1b.** Force estimated by considering the arm as a rigid body | | | | | |
| --- | --- | --- | --- | --- | --- |
| **Term** | **Estimate** | **SE** | **χ^2^** | ***df*** | ***P*** |
| Intercept | 7.478 | 0.249 | ^(1)^ | ^(1)^ | ^(1)^ |
| Hammer type (H) | -0.713 | 0.049 | 40.21 | 1 | < 0.001 |
| Age | -0.020 | 0.012 | 2.406 | 1 | 0.12 |
| ^(1)^ not shown because of having a very limited interpretation | | | | | |

**Fig. S2** Lifting force for the naïve condition for natural (N) and hollowed (H) hammers estimated by ignoring the weight of chimpanzees arms (a) or by considering each arm as a rigid body rotating about the shoulder (b).

**Supplementary References**

Grützmacher, K., Keil, V., Leinert, V., Leguillon, F., Henlin, A., Couacy‐Hymann, E., ... & Leendertz, F. H. (2017). Human quarantine: Toward reducing infectious pressure on chimpanzees at the Taï Chimpanzee Project, Côte d'Ivoire. *American journal of primatology*. DOI 10.1002/ajp.22619

Schoonaert K, D’Août K, Aerts P (2007) Morphometrics and inertial properties in the body segments of chimpanzees (*Pan troglodytes*). J Anat 210:518-531

Günther MM, Boesch C (1993) Energetic cost of nut-cracking behaviour in wild chimpanzees. In: Prueschoft H, Chivers D (eds) Hands of primates. Springer, Vienna, pp 109-129
